# Distinct Expression Profiles of Three Melatonin Receptors during Early Development and Metamorphosis in the Flatfish *Solea senegalensis*

**DOI:** 10.3390/ijms151120789

**Published:** 2014-11-13

**Authors:** Olivier Lan-Chow-Wing, Francesca Confente, Patricia Herrera-Pérez, Esther Isorna, Olvido Chereguini, Maria del Carmen Rendón, Jack Falcón, José A. Muñoz-Cueto

**Affiliations:** 1 Department of Biology, Faculty of Marine and Environmental Sciences, University of Cádiz, Marine Campus of International Excellence (CEIMAR), Agrifood Campus of International Excellence (ceiA3), E-11510 Puerto Real, Spain; E-Mails: francesca.confente@uca.es (F.C.); patricia.herrera@uca.es (P.H.-P.); maricarmen.rendon@uca.es (M.C.R.); 2 Department of Physiology (Animal Physiology II), Faculty of Biology, Complutense University of Madrid, E-28040 Madrid, Spain; E-Mail: eisornaa@bio.ucm.es; 3 IEO, Spanish Institute of Oceanography, Santander Oceanographic Centre, Promontorio de San Martín, s/n, P.O. Box 240, E-39080 Santander, Spain; E-Mail: o.chereguini@st.ieo.es; 4 Aragó Laboratory—UMR7628 (CNRS and UPMC) and GDR2821 (CNRS/Ifremer), F-66651 Banyuls/Mer, France; E-Mail: falcon@obs-banyuls.fr; 5 INMAR-CACYTMAR Research Institutes, Puerto Real University Campus, E-11510 Puerto Real, Spain

**Keywords:** melatonin receptors, development, metamorphosis, flatfish

## Abstract

Melatonin actions are mediated through G protein-coupled transmembrane receptors. Recently, *mt1*, *mt2*, and *mel1c* melatonin receptors were cloned in the Senegalese sole. Here, their day-night and developmental expressions were analyzed by quantitative PCR. These results revealed distinct expression patterns of each receptor through development. *mel1c* transcripts were more abundant in unfertilized ovulated oocytes and declined during embryonic development. *mt1* and *mt2* expression was higher at the earliest stages (2–6 days post-fertilization), decreasing before (*mt2*) or during (*mt1*) metamorphosis. Only *mt1* and *mel1c* expression exhibited day-night variations, with higher nocturnal mRNA levels. These results suggest different roles and transcriptional regulation of these melatonin receptors during flatfish development and metamorphosis.

## 1. Introduction

In fish, as in other non-mammalian vertebrates, the pineal organ transduces the time of the day and of the year into a rhythmic melatonin secretion, which is modulated by environmental light and temperature [1,2]. This hormone has been involved in many circadian and circannual rhythmic processes such as reproduction and feeding [2]. Melatonin actions are mediated through seven-transmembrane domain G protein-coupled receptors [3]. Three high-affinity melatonin receptor subtypes have been cloned in fish: *mt1*, *mt3*, and *mel1c* [4]. These receptors seem to be widely distributed through the brain but also in several peripheral tissues such as liver, gills, and skin [4,5].

Melatonin and the pineal gland seem to play an important role in the early development of fish [6,7]. Indeed, the pineal gland develops before the retina, both structurally and functionally: morphologically identified photoreceptor cells, photopigment molecules, pinealofugal innervation and melatonin biosynthesis all start in the pineal organ, well before the retina [8,9,10]. It has been proposed that melatonin mediates phototransduction during hatching [6,9]. In anurans, the hormone has been implicated in metamorphosis, acting as a transducer of environmental information that modulates the timing of this event [11]. It has been suggested that the hormone antagonizes the hypothalamus-pituitary-thyroid axis, because thyroid hormones and melatonin are negatively correlated [11] and ocular melatonin binding sites decrease during amphibian metamorphosis [12]. However, data on the exact role melatonin plays during development remain anecdotal [6,13], and absolutely no information is available in metamorphic fish species. For this reason, and because we are accumulating data on the melatonin-synthesizing process in the Senegalese sole, *Solea senegalensis* [14,15,16,17], we decided to investigate the developmental pattern of the melatonin receptors that we have previously cloned in this nocturnal flatfish [18].

## 2. Results

The day-night expression of the three melatonin receptors was examined in developing sole by quantitative real time PCR, using *β-actin* for normalization. Expression of *β-actin* did not exhibit significant day-night differences at the ontogenetic stages analyzed (*p* values from 0.054 to 0.92), revealing that it represented an adequate housekeeping gene for this study.

### 2.1. mt1 Expression

**Figure 1:**
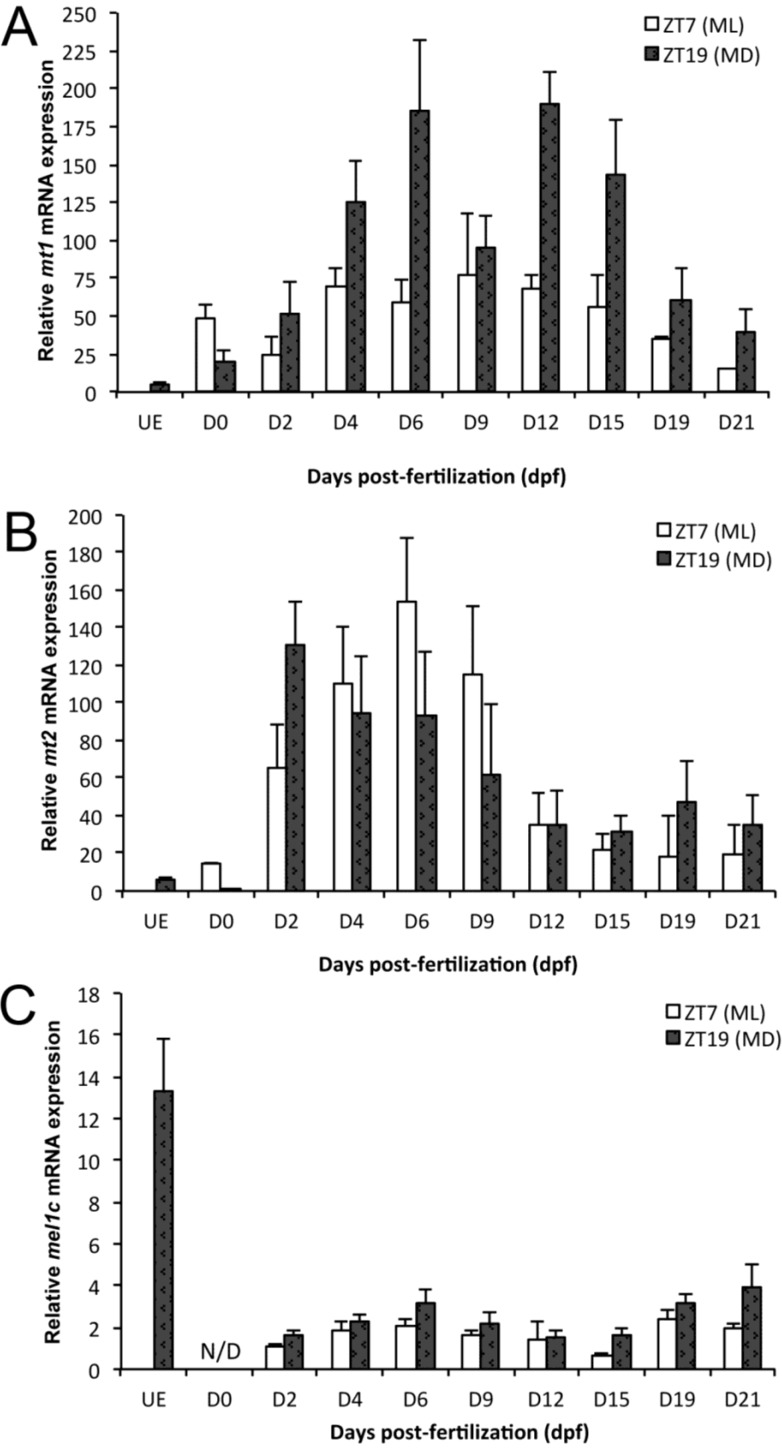
Relative expression of *mt1* (**A**); *mt2* (**B**); and *mel1c* (**C**) in unfertilized eggs (UE), embryos, and larvae at different stages of development in Senegalese sole specimens sampled at ZT7 (midday or ML) and ZT19 (midnight or MD). mRNA levels were measured by real-time PCR on pools of animals. Data are shown as the mean ± SEM (*n* = 3–4). For statistical analysis, see Material and Methods and Table 1.

Both developmental and day-night variations in *mt1* expression were revealed by two-way ANOVA analysis of the data obtained. Transcript levels were higher at night; also, a significant interaction between developmental stage and hour of sampling was found (Table 1). Thus, a Kruskal–Wallis one-way analysis of variance was performed, revealing that significant developmental changes in *mt1* mRNA levels were evident mainly at night (Figure 1A, Table 1). *mt1* transcripts were very low in unfertilized eggs and augmented significantly in 0 dpf embryos. Its expression increased again between 0 and 6 dpf (9-fold), remained elevated from 6 to 15 dpf (early metamorphosis), decreasing thereafter to 19 dpf (metamorphic climax) and 21 dpf (late metamorphosis). Although *mt1* relative expression was elevated at night in relation to day-time levels on almost all sampling days, differences were only significant at 6 and 12 dpf (Figure 1A, Table 1).

**Table 1:** Significance levels of statistical analysis for each melatonin receptor.

***mt1***																														
**TWO-WAY ANOVA**																							**KRUSKAL WALLIS**							
Developmental stage		Hour of sampling														Interaction							H = 60.4; *p* < 0.0001							
F = 10.83; *p* < 0.001		F = 23.6; *p* < 0.001														F = 3.15; *p* < 0.01														
**BONFERRONI TEST**																														
Developmental stage		UE		0 dpf			2 dpf			4 dpf				6 dpf				9 dpf			12 dpf				15 dpf		19 dpf		21 dpf	
Hour of sampling		MD		ML		MD	ML		MD	ML		MD		ML			MD	ML		MD	ML			MD	ML	MD	ML	MD	ML	MD
Comparison		a		abcd		ab	ab		abcd	abcd		cde		abcd			e	abcd		bcde	abcd			e	abcd	de	abc	abcd	ab	abc
***mt2***																														
**TWO-WAY ANOVA**																							**KRUSKAL WALLIS**							
Developmental stage		Hour of sampling											Interaction										H = 57.7; *p* < 0.0001							
F = 10.67; *p* < 0.001		F = 0.10; *p* = 0.756											F = 2.29; *p* < 0.05																	
**BONFERRONI TEST**																														
Developmental stage		UE		0 dpf			2 dpf			4 dpf				6 dpf				9 dpf				12 dpf			15 dpf		19 dpf		21 dpf	
Hour of sampling		MD		ML		MD	ML		MD	ML		MD		ML			MD	ML		MD		ML		MD	ML	MD	ML	MD	ML	MD
Comparison		a		abc		a	abcdefg		fg	defg		cdefg		g			bcdefg	efg		abcdefg		abcdef		abcdef	abcde	abcde	abcd	abcdef	abcd	abcde
***mel1c***																														
**TWO-WAY ANOVA**																														
Developmental stage	Hour of sampling												Interaction																		
F = 6.54; *p* < 0.001	F = 16.87; *p* < 0.001												F = 0.44; *p* = 0.8729																		
**NEWMAN-KEULS TEST**																															
Developmental stage	UE				0 dpf			2 dpf			4 dpf				6 dpf				9 dpf			12 dpf			15 dpf		19 dpf		21 dpf		
Comparison	a				N/D			bc			bc				b				bc			c			c		b		b		
Hour of sampling	ML		MD																												
Comparison	a		b																												

There are no statistical differences among groups that share common letters. UE: unfertilized eggs; ML midday or ZT7; MD midnight or ZT19; N/D: non detected, mRNA levels below the limits of detection of the technique.

### 2.2. mt2 Expression

*mt2* expression varied during development, without significant day-night changes (two-way ANOVA, Figure 1B, Table 1). There was a significant interaction between the hour of sampling and developmental stage, and differences among experimental groups were also significant as revealed by a Kruskal–Wallis one-way analysis of variance (Table 1). *mt2* transcripts were found at low levels in unfertilized eggs and at 0 dpf embryos. Diurnal *mt2* mRNA levels increased 10-fold from 0 to 6 dpf and then decreased 10-fold between 6 to 15 dpf, remaining low until 21 dpf (Figure 1B). Nocturnal *mt2* expression exhibited a significant 500-fold rise between 0 to 2 dpf, followed by a progressive reduction (4.5-fold) from 2 dpf until the end of metamorphosis (Figure 1B).

### 2.3. mel1c Expression

*mel1c* transcript levels were abundant in unfertilized eggs and decreased considerably after fertilization, being below the limits of detection at 0 dpf (Figure 1C). Significant developmental and day-night fluctuations were detected, with higher transcript levels at night than during the daytime (Table 1). No interaction was found between the two factors (Table 1). *mel1c* expression doubled from 2 to 6 dpf, then decreased 2-fold to 15 dpf, only to increase again to 21 dpf (Figure 1C, Table 1).

## 3. Discussion

We reported here, for the first time in fish, distinct day-night and developmental expression patterns of the genes encoding for three different melatonin receptor subtypes (*mt1*, *mt2*, and *mel1c*).

In the sole, the three melatonin receptor mRNAs were detected in unfertilized ovulated oocytes. *mel1c* transcripts were abundant in unfertilized eggs, and decreased at early stages of development, whereas the transcription of the other two receptor genes, *mt1* and *mt2*, was low in unfertilized eggs and increased after fertilization. Similar results were reported in the Japanese quail, *Coturnix japonica* [19,20]. This expression reflects the presence of mRNA from maternal origins, and it has been proposed that such receptors could mediate some protective effects of melatonin from free radicals formed during intensive embryonic metabolism [19,20]. Interestingly, a very marked nocturnal spawning rhythm was reported in sole, with spawning beginning after dusk and the acrophase occurring around 23 h [21]. Whether these *mel1c* receptors are mediating the synchronizing effects of melatonin on sole spawning remains to be investigated.

**Figure 2:**
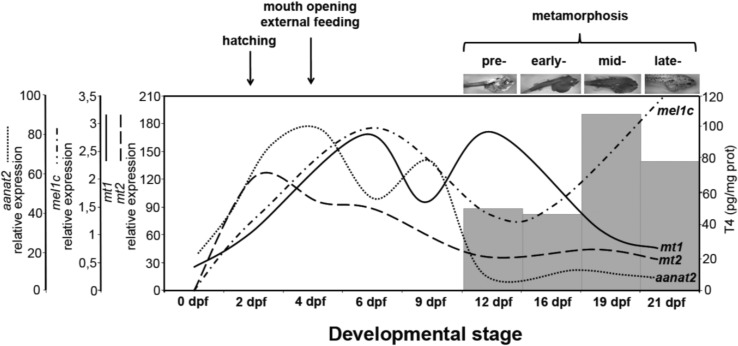
Developmental expression profiles of the three melatonin receptor subtypes (*mt1*, *mt2*, *mel1c*) and the pineal-specific enzyme arylalkylamine *N*-acetyltransferase (*aanat2*) [16], together with thyroid hormone levels (T4, grey columns) [25] in sole. Note that the melatoninergic system is up-regulated at hatching and the onset of external feeding; also note the inverse profile between *mt1*, *mt2*, and *aanat2* expression and thyroid hormone levels during sole development and metamorphosis.

At hatching (*i.e.*, 2 dpf) a strong increase in *mt2* transcript levels was initiated, whereas the elevation in *mt1* mRNA levels occurred from 0 dpf but becomes more conspicuous at 4 dpf, coinciding with the opening of the mouth and beginning of external feeding (Figure 2). This is concomitant with the onset of pineal (2 dpf) and retinal (3 dpf) photoreception, elevation of pineal *aanat2* (2 dpf) and retinal *aanat1a* (4 dpf) mRNA levels [16,17,22], and, probably, a rise in circulating melatonin. Recently, it has been demonstrated that lighting conditions during incubation affect hatching rhythms and subsequent larval development (growth, mouth opening, and appearance of pectoral fins) in the Senegal sole [23]. Expression of melatonin receptors (particularly *mt2*), is up-regulated in *Danio rerio* embryos, where Mt2 might play a major role in mediating melatonin-accelerated development at these early stages [6]. Indeed, melatonin stimulated cell proliferation in zebrafish, and melatonin-treated embryos developed faster and hatched earlier, the maximal effects being obtained when melatonin receptors were widely expressed [6]. In another flatfish, the halibut (*Hippoglossus hippoglossus*)*,* hatching seems to be modulated by photoperiod acting through the pineal gland [9]. The putative role of *mt1* and *mt2* receptors in the mediation of photoperiod effects reported on sole feeding and hatching [23,24] rhythms requires further characterization.

An interesting finding of this study is that different day-night and developmental patterns of expression exist for the three melatonin receptor subtypes. *mt1* mRNA abundance exhibited day-night variation, with higher nocturnal expression. In agreement with these results, melatonin-synthesizing enzymes *aanat1a* and *aanat2* also exhibited higher transcript levels at MD than at ML from 2 dpf and at most developmental stages analyzed [16,17]. The lack of midday/midnight difference in *mt2* mRNA abundance may indicate either an absence of daily rhythm or a phase difference between the *mt1* and *mt2* mRNA rhythms. Otherwise, *mt2* daily rhythms of expression may be masked by phase differences in transcript levels between different expression sites.

In flatfish (as in anurans), thyroid hormones drive morphological, molecular, and physiological changes that occur during metamorphosis [26]. In addition, the thyroid system (hormone levels, receptor expression, deiodinase expression and activity) is up-regulated during sole metamorphosis [25]. We now provide evidence that the melatonin system is concomitantly down-regulated, as summarized in Figure 2. This includes the melatonin-synthesizing enzymes *aanat1* and *aanat2* [16,17], and the melatonin receptors *mt1* and *mt2* (this study). Earlier studies indicated that melatonin is involved in amphibian metamorphosis, acting as an antagonist of thyroid hormones [11]; similar antagonizing effects were also reported in some non-metamorphic fish [27], but such antagonism remains unexplored in flatfish.

The *mel1c* receptor subtype was cloned for the first time from isolated melanophores of *Xenopus* [28] and seems to mediate melatonin effects on pigmentation [29]. It is interesting to note that photoperiod and light spectrum affect early growth and development in sole, including eye pigmentation [23]. In the adult sole, *mel1c* expression was seen in the retina, brain, pituitary, muscle, and skin [18]. The skin of the sea bass *Dicentrarchus labrax* [4] and goldfish scales [30] also express *mel1c*. Poikilotherms, including fish, exhibit circadian rhythms in color change, with nocturnal blanching related to pigment melanophore aggregation induced by melatonin [31]. As in other flatfish, pigmentation abnormalities have been observed during sole metamorphosis, which appears to be influenced by light intensity and background color of tanks during larval rearing [32]. Interestingly, *mel1c* expression exhibited day-night differences during sole development, with higher nocturnal levels, as reported for *aanat1a* and *aanat2* in developing sole [16,17]. Moreover, regulation of *mel1c* expression during development appears to differ compared to *mt1* and *mt2* because its expression increased throughout metamorphosis. In the course of this event, the right side of the body (ocular side) becomes more pigmented and the left side (blind side) loses coloration and becomes almost entirely white. Thus, *mel1c* receptors expressed during development could be mediating early effects of photoperiod and/or melatonin on sole pigmentation. 

## 4. Materials and Methods

### 4.1. Animals and Sampling

Senegalese sole fertilized eggs were obtained in May (0 days post-fertilization or 0 dpf) from “IFAPA El Toruño” (Junta de Andalucía, Puerto de Santa María, Spain) and maintained in the “Laboratorio de Cultivos Marinos” (University of Cádiz) as previously described [16,25]. Eggs were incubated under a natural photoperiodic environment (sunrise 07:31 h, sunset 21:22 h, GMT + 2, illumination of 300-500 lux on water surface), at 19 ± 1 °C of temperature and 39 ppt of salinity. Animals were sampled at nine stages of development: before hatching (0 dpf), before the beginnings of metamorphosis (2, 4, 6, 9 dpf), and during metamorphosis (pre-, early-, middle-, and late-metamorphosis; 12, 15 19 and 21 dpf, respectively). At each developmental stage, pools of whole animals were obtained at ZT7 (midday, 14:30 h GMT + 2) and ZT19 (midnight, 02:30 h GMT + 2), which exhibited day-night differences in the expression of melatonin-synthesizing enzymes [16,17]. The number of individuals per pool was 20–30 at 0–6 dpf, 10–20 at 9–15 dpf, and 5–10 at 19–21 dpf, respectively. The larvae’s metamorphic process was followed daily under a photomicroscope. Senegalese sole ovulated unfertilized eggs were obtained from the IEO (MICINN, Centro Oceanográfico de Santander, Santander, Spain) and used to detect the presence of melatonin receptor mRNA of maternal origin. Samples were frozen in liquid nitrogen and stored at −80 °C until used. All animal experiments were approved by the Institutional Animal Care and Use Committee at the University of Cádiz and were conducted in accordance with international standards.

### 4.2. Quantitative Real-Time PCR Analysis

Total RNA was extracted from larval pools using “EUROzol” (EuroClone, Siziano, Italy) according to the manufacturer’s instructions. Total RNA (1 μg) was reverse-transcribed and genomic DNA removed (QuantiTect^®^ Reverse Transcription Kit, Qiagen, Hilden, Germany). Real-time gene expression analysis was performed in a Chromo 4™ Four-Color Real-Time System (Bio-Rad, Alcobendas, Spain), using *β-actin* for normalization (GeneBank accession number DQ485686). PCR reactions were developed in duplicate in a 25 μL volume using cDNA generated from 1 μg of RNA, iTaq™ SYBR^®^ Green Supermix with ROX (Bio-Rad), and specific primers (0.4 μM, Table 2) designed from the cloned *mt1*, *mt2*, and *mel1c* sequences of the sole [18]. All calibration curves exhibited slopes close to −3.32 and efficiencies around 100%. The conditions of the PCR reactions were similar for the four genes analyzed (38 cycles): 3 min at 95 °C, 30 s at 95 °C, 45 s at 60 °C, and 45 s at 72 °C. PCR products were run in agarose gels, sequenced, and melting curves were analyzed for each sample to confirm that only a single sequence was amplified. Negative controls included replacement of cDNA by water and the use of non retro-transcribed total RNA. The ΔΔ*C*t method [33] was used to determine the relative mRNA expression.

**Table 2:** Sequences of the primers used.

Primer Name	Sequence (5'-3')
*ssmt1F*	GCGGAAAGGAATAAATGAGGC
*ssmt1R*	GGAGTTGGTGCGTCACAGTG
*ssmt2F*	GCGTCAACGAAGAGCGAAAT
*ssmt2R*	GCCCGAAACTGGCCATAAAT
*ssmel1cF*	ACTTCAACAGCTGCCTCAACG
*ssmel1cR*	AGCAAACGTGGGATGCAAAG
*ssβactinF*	GGATCTGCATGCCAACACTG
*ssβactinR*	TCTGCATCCTGTCAGCAATG

### 4.3. Statistical Analysis

Day-night statistical differences in housekeeping gene expression in different developmental stages were determined by Paired T-test. Developmental and day-night statistical differences among groups were analyzed using two-way ANOVA. When significant interaction between the hour of the day and developmental stage was found (*i.e.*, for *mt1* and *mt2*), differences among groups were determined using non-parametric Kruskal–Wallis analysis followed by Bonferroni test because of the absence of homogeneous variance even after data transformation. When no significant interaction between factors existed (*i.e.*, for *mel1c*), each factor was analyzed separately by one-way ANOVA followed by the Newman–Keuls test. Statistical tests were made using the Statgraphics plus (version 5.1) software (Manugistics, Rockville, MD, USA, 2000).

## 5. Conclusions

Taken together, all these data strengthen the idea that melatonin, acting through Mt1, Mt2, and/or Mel1c receptors, could play an important role in setting physiological and behavioral rhythms during sole gametogenesis, development, and metamorphosis. The presence of high *mel1c* transcript levels in mature unfertilized eggs suggests that this receptor could be mediating melatonin effects in ovulated oocytes. Moreover, we have demonstrated that these receptors exhibited distinct developmental expression profiles, suggesting that they are subjected to different transcriptional regulatory mechanisms. The fact that *mt1* and *mt2* receptor expression decline during metamorphosis, when thyroid hormone levels rise, reinforces the interest of analyzing the functional antagonism between melatoninergic and thyroid hormone system in the regulation of this event. Further studies are being directed to elucidate the melatonin effects on relevant processes such as cell proliferation, hatching, metamorphosis, locomotor activity, and feeding at early development stages of the Senegalese sole, and to determine which melatonin receptors appear involved in these putative actions.
